# Case Report: Acute kidney injury due to minimal dilated obstructive nephropathy in the context of gastric cancer

**DOI:** 10.3389/fonc.2025.1586443

**Published:** 2025-09-01

**Authors:** Xinyu Xiang, Xiaobao Wei, Jiayi Miao, Meng Sun, Wei Cao, Baiqiao Zhao, Yiwen Zhang, Juanyu Wei, Lin Zhu, Yongping Zhang, Ting Zhang, Liyuan Zhang

**Affiliations:** ^1^ Department of Nephrology, Lianyungang Clinical College of Nanjing Medical University, Lianyungang, China; ^2^ Department of Nephrology, The First People’s Hospital of Lianyungang, Lianyungang, China; ^3^ Department of Nephrology, The Affiliated Lianyungang Hospital of Xuzhou Medical University, Lianyungang, China

**Keywords:** acute kidney injury, gastric cancer, mild hydronephrosis, obstructive nephropathy, peritoneal metastasis 1

## Abstract

Postrenal obstruction is a rare but reversible cause of acute kidney injury (AKI), often underrecognized when hydronephrosis is mild or absent. We present a 55-year-old woman with gastric cancer who developed severe AKI requiring hemodialysis. Initial non-contrast abdominal CT revealed only mild bilateral hydronephrosis without obvious ureteral obstruction. Given these subtle radiologic findings and a history of chemotherapy and NSAID exposure. Initially, a multidisciplinary team attributed the AKI to intrinsic renal causes. Subsequent renal biopsy revealed only minimal glomerular changes, insufficient to explain the degree of renal dysfunction. Despite supportive care, her renal function continued to decline. Further urological evaluation led to the placement of bilateral ureteral stents, which resulted in a prompt increase in urine output and improvement in serum creatinine. However, rapid restenosis occurred within four days, necessitating percutaneous nephrostomy. This two-step intervention restored renal function and improved short-term prognosis. This case underscores the diagnostic challenge of postrenal AKI in malignancy, particularly when imaging findings are subtle. Peritoneal carcinomatosis may cause ureteral encasement through mechanisms such as inflammation, fibrosis, and lymphatic disruption, often without significant collecting system dilation. Timely urologic intervention, guided by clinical judgment and supported by multidisciplinary collaboration, is critical to improving outcomes in such atypical presentations.

## 1 Introduction

Postrenal AKI is caused by urinary tract obstruction, accounts for approximately 5%–10% of AKI cases and is typically reversible if identified early (1, 2). However, diagnosis becomes challenging when imaging reveals only mild hydronephrosis or no overt signs of obstruction. In patients with gastric cancer, AKI is more frequently attributed to chemotherapy-induced nephrotoxicity, sepsis, or hemodynamic instability (3–6). Obstructive etiologies, particularly those related to peritoneal metastasis (PM), are rare and often underrecognized (7). Previous reports have more commonly linked malignant urinary tract obstruction to direct invasion or metastasis involving the bladder (8) or cervix (9, 10). Recent studies indicate that in malignancy-associated obstruction—especially when caused by extrinsic ureteral compression due to peritoneal spread or fibrosis—the collecting system may not dilate significantly. This obstructive urinary tract lesion with minimal dilation of the collection system is associated with delayed diagnosis and poor renal prognosis (11–13). Here, we present a rare case of postrenal AKI in a patient with gastric cancer, in whom peritoneal metastasis was considered. The initial imaging demonstrated only mild bilateral hydronephrosis. Despite the delay in diagnosis, subsequent urological interventions led to full recovery of renal function. This case underscores the importance of maintaining a high index of suspicion and re-evaluating the diagnosis in patients with malignancy and AKI.

## 2 Case presentation

### 2.1 Initial presentation

**Figure 1:**
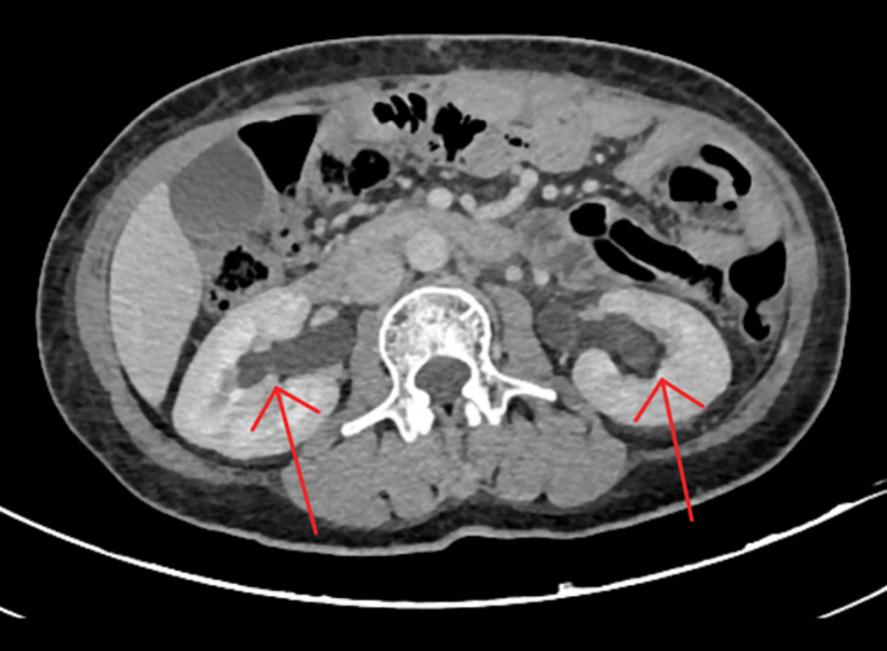
Non-contrast abdominal CT scan revealing minimal bilateral hydronephrosis. The scan showed mild dilation of the bilateral renal pelvis and collecting systems, without visible ureteral stones, wall thickening, strictures, or other obstructive lesions. These subtle findings likely contributed to the initial underestimation of the severity of urinary tract obstruction.

A 55-year-old woman presented with nausea, back pain, and oliguria. One year prior, she had undergone total gastrectomy for gastric adenocarcinoma (T4N2M0, stage IIIA), followed by eight cycles of adjuvant chemotherapy with oxaliplatin and capecitabine, which concluded six months earlier. She had no other significant medical history. Five days before admission, she experienced recurrent flank pain and sought emergency care. Abdominal ultrasonography revealed bilateral hydronephrosis with proximal ureteral dilation and no residual bladder urine (There is separation of the echogenic band in the renal collecting systems, measuring approximately 17 mm on the right side and 11 mm on the left side.). Non-contrast abdominal CT scan showed mild bilateral hydronephrosis, perihepatic and perisplenic fluid accumulation, and localized intestinal distension, without evidence of soft tissue masses in the abdomen or retroperitoneum (**Figure 1**). Oral analgesics provided symptomatic relief, but the patient declined further diagnostic workup.

### 2.2 Diagnostic workup

On admission, her vital signs were stable: T 36.5°C, P 86 beats/min, R 16 breaths/min, BP 124/78 mmHg. Physical examination revealed mild pitting edema in both lower extremities. Laboratory findings revealed stage 3 AKI according to the Kidney Disease: Improving Global Outcomes (KDIGO) criteria, accompanied by hyperkalemia: serum creatinine 1099.9 μ mol/L (baseline: 42.9 μ mol/L), and potassium 6.85 mmol/L. On admission, the patient presented with anuria; urine analysis was deferred due to anuria. Emergency hemodialysis was initiated via a temporary femoral venous catheter, along with bladder catheterization. Post-dialysis, nausea improved, potassium normalized, and serum creatinine decreased to 998.9 μ mol/L. Due to persistent anuria and modest improvement in renal function, additional hemodialysis was performed on days 3 and 5.

### 2.3 Interventions and management

**Figure 2:**
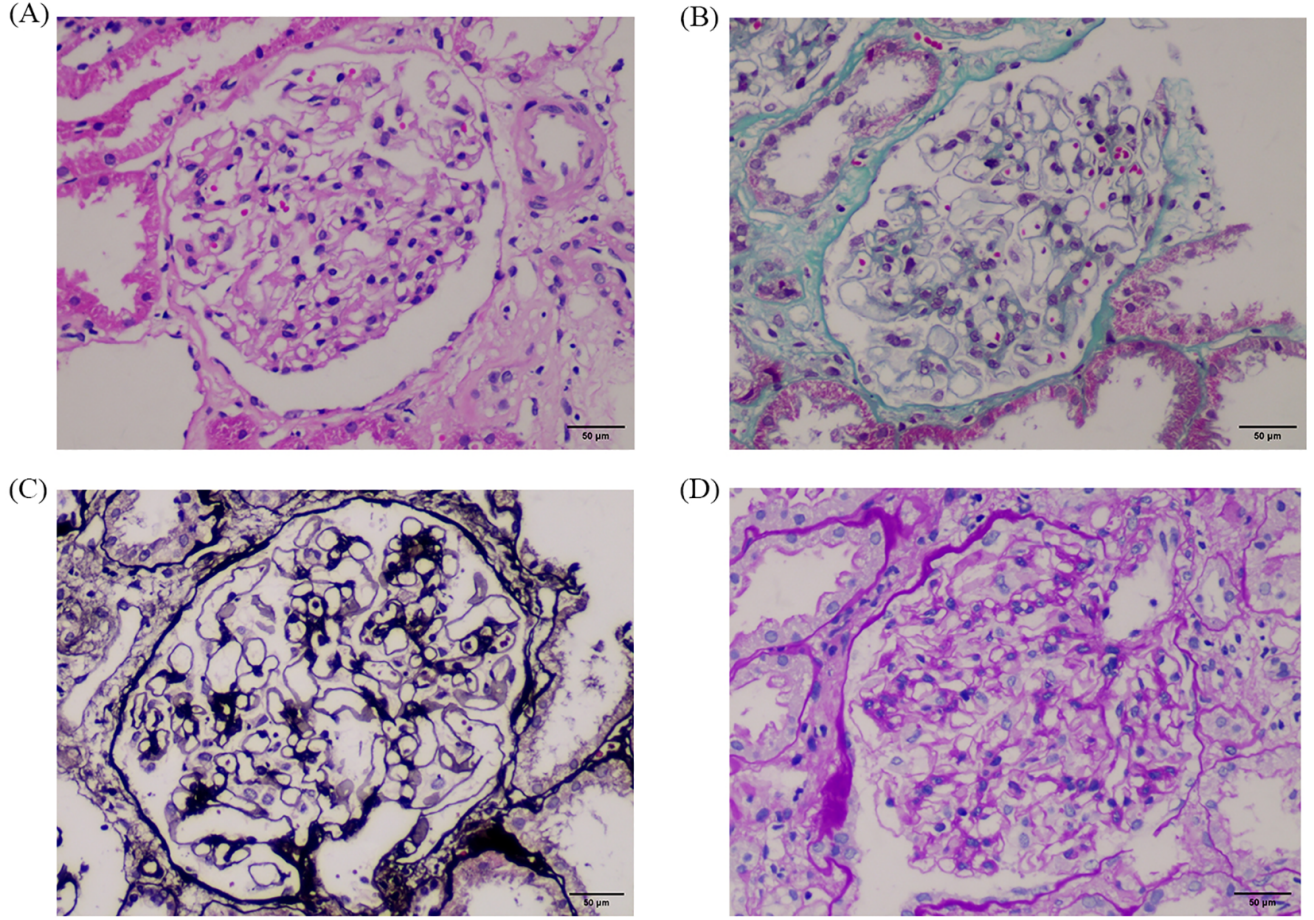
Renal biopsy findings at 400× magnification. Panels show results of different staining techniques: **(A)** H.E, **(B)** Masson, **(C)** P.A.S.M, and **(D)** P.A.S. The glomeruli exhibit only minimal pathological changes, with no segmental sclerosis, crescents, or necrosis. Tubules show no evidence of necrosis or cast formation. These findings support a diagnosis of minimal glomerular lesion without intrinsic renal injury, consistent with a postrenal cause of acute kidney injury. A representative 50 μm scalebar was added using ImageJ based on a 400× magnification and standard field-of-view estimation.

On day 7, a multidisciplinary team (nephrology, urology, oncology, and radiology) convened to review the case. Given the mild hydronephrosis and history of nephrotoxic chemotherapy, intrinsic AKI was initially prioritized. Obstruction was reconsidered only after biopsy excluded tubular injury. Corticosteroids (40 mg prednisone daily) were started to reduce inflammation and optimize biopsy safety. On day 9, ultrasound-guided renal biopsy was performed without complications. Despite ongoing hemodialysis and symptomatic treatment, on day 13, the renal biopsy pathology showed only minor glomerular abnormalities (**Figure 2**). Repeat imaging revealed worsening bilateral hydronephrosis (There is separation of the echogenic band in the renal collecting systems, measuring approximately 25 mm on the right side and 24 mm on the left side), prompting a urology consultation. On day 14, retrograde ureteral stenting (RUS) was performed. Within 24 hours, urine output increased to 4850 mL, lower extremity edema improved. Dialysis was discontinued on day 16 following renal recovery (serum creatinine: 155.6 μ mol/L), and the catheter was scheduled for removal. On day 17, oliguria recurred and furosemide administration had no effect. A plain abdominal X-ray confirmed proper stent placement, but persistent hydronephrosis suggested ongoing obstruction. On day 21, percutaneous nephrostomy (PCN) was performed under CT guidance, yielding 3000 mL of urine output within 24 hours. Over the next few days, renal function, serum creatinine, and electrolytes normalized. The femoral catheter was removed on day 24, and the patient was discharged in stable condition on day 26.

### 2.4 Follow-up

The patient underwent cancer-specific follow-up in the oncology department every week after discharge. Twenty-one days after discharge, the patient came to our hospital to reinsert the double-J tube due to the loss of the left nephrostomy tube. During the 2-month follow-up period, the patient’s stent remained unobstructed without any signs of failure (creatinine was within the normal range, and imaging showed that hydronephrosis was resolved). Unfortunately, approximately two months after discharge, the patient visited another hospital due to persistent abdominal pain, abdominal distension and other symptoms. During the surgical treatment of intestinal obstruction, multiple metastatic lesions in the abdominal and pelvic cavities were found, marking significant tumor progression. She refused further diagnosis or treatment procedures and chose to relieve her symptoms with sustained-release oxycodone (5 milligrams every 12 hours). Reviewing the case, the diagnosis of peritoneal metastasis in the patient was based on reasonable clinical suspicion (Imaging suggests indirect signs of peritoneal metastasis), multidisciplinary team (MDT) discussions, and subsequent disease evolution.

**Figure 3:**
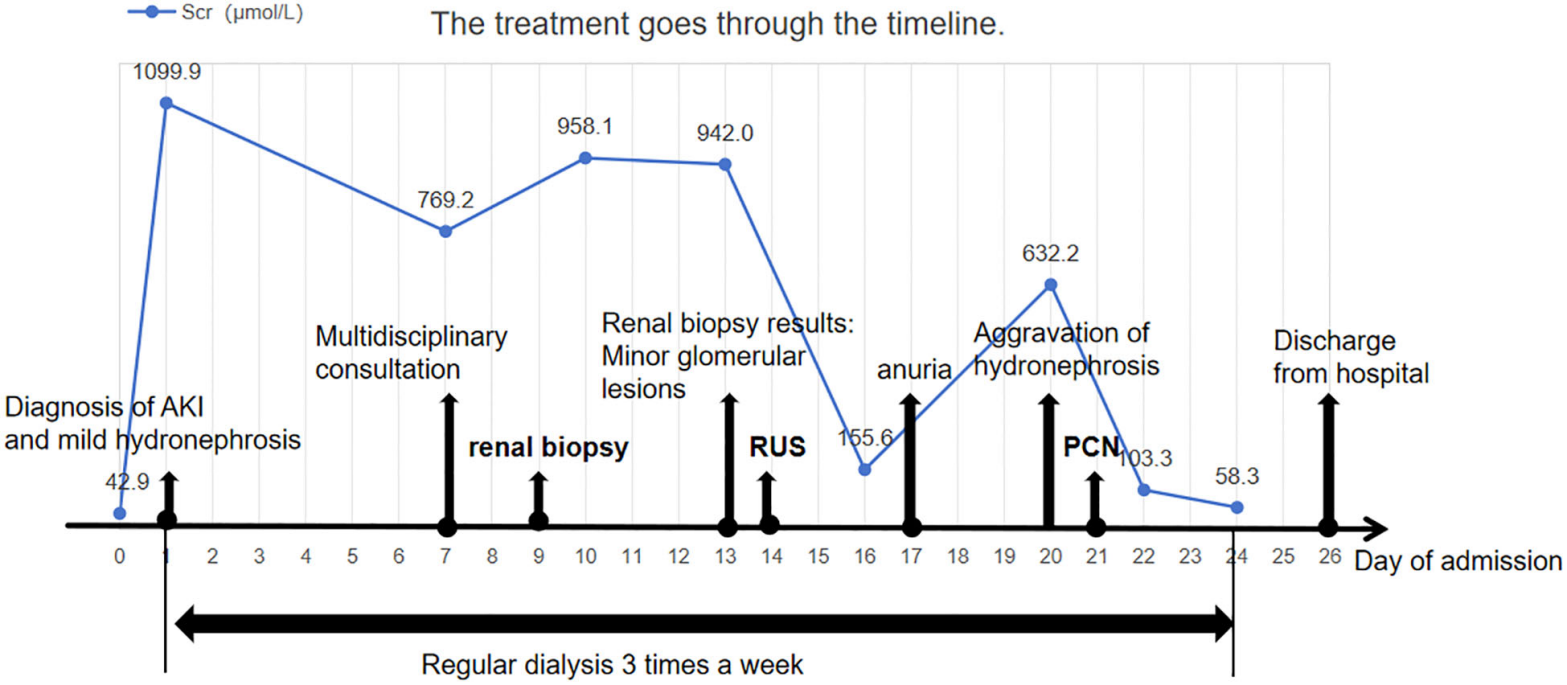
Clinical timeline of the patient’s presentation, diagnosis, and management. Timeline summarizing key clinical events, including serial serum creatinine levels, timing of major interventions (renal biopsy, retrograde ureteral stenting, and percutaneous nephrostomy), dialysis sessions, and clinical milestones such as onset of anuria and hospital discharge. The figure illustrates how initially subtle imaging findings contributed to a delay in diagnosis. Early recognition and prompt relief of obstruction may improve short-term outcomes in patients with malignancy-associated obstructive AKI.

We have created a timeline illustrating the patient’s clinical course and management during hospitalization (**Figure 3**).

### 2.5 Patient’s point of view

The patient reported significant anxiety during the period of diagnostic uncertainty. After inviting a multidisciplinary consultation and engaging in thorough communication, she adopted a positive attitude towards renal biopsy. Once the obstruction was confirmed, we informed her of the likelihood of persistent renal dysfunction and potential disease progression. She and her family clearly expressed a desire for active but minimally invasive management to preserve kidney function, avoid ICU-level care, and maintain quality of life for as long as possible. She expressed gratitude for the multidisciplinary care she received but stated her wish for earlier detection of obstruction in similar cases.

## 3 Discussion

### 3.1 Mechanisms of peritoneal metastasis-associated obstructive nephropathy

In cancer patients, uric acid crystals in tumor lysis syndrome, light chain casts in multiple myeloma, or the crystallization of certain drugs such as platinum-based chemotherapy agents can lead to tubular obstruction in the kidneys (7, 14, 15). Tumor cells may also directly extend into and invade both ureters or renal parenchyma, causing local tissue destruction (6, 16). In rare cases, treatment-related fibrosis from radiotherapy or chemotherapy (17–19), as well as peritoneal metastasis (9), can result in external compression of the urinary tract, as seen in our patient, contributing to the development of acute kidney injury.

Peritoneal metastasis is a common complication in patients with gastric cancer, observed in approximately 10%-20% of cases at the time of initial diagnosis (20–22). This proportion may be even higher in surgical and observational studies (23, 24). Cancer cells can spread into the abdominal cavity through direct invasion or hematogenous dissemination, implanting on the peritoneal surface and forming peritoneal metastases (25). As metastases progress, tumor cells can exert pressure on nearby structures, like the ureter, due to local growth and expansion. This pressure can impair urinary flow and lead to obstruction. Molecular drivers play pivotal roles in ureteral compression from gastric cancer-related PM. Metastatic lesions release abundant proinflammatory cytokines, especially interleukin-6 (IL-6), which activates the JAK/STAT3 pathway and recruits macrophages and neutrophils. This process fosters a proinflammatory milieu that promotes intra-abdominal adhesions and sets the stage for subsequent fibrosis (26–28). Both tumor cells and infiltrating macrophages secrete high levels of transforming growth factor-β (TGF-β), which bind TGFBR1/2 and triggers phosphorylation of Smad2/3. The Smad2/3-Smad4 complex then translocates to the nucleus to initiate transcription of profibrotic genes such as COL1A1, fibronectin (FN1), and connective tissue growth factor (CTGF), leading to excessive extracellular matrix (ECM) deposition around the ureters and the development of peritoneal adhesions (27, 29–31). Vascular endothelial growth factor-A (VEGF-A), produced by tumor cells, binds VEGFR2 on endothelial cells, thereby activating the PI3K/Akt and ERK1/2 pathways to promote aberrant angiogenesis. Additionally, VEGF induces phosphorylation of VE-cadherin, which disrupts tight junctions and increases vascular permeability. The resulting vascular leakage, combined with localized edema and tissue congestion, further intensifies pressure on surrounding structures including the ureters (32–35). Moreover, metastasized peritoneal tumor cells can disrupt lymphatic drainage in the abdominal cavity, which contributes to increased intra-abdominal pressure and aggravates urinary tract obstruction (21, 36–39). Recent studies have discovered several biomarkers closely related to peritoneal metastasis of gastric cancer. CLDN18.2 (40), highly expressed in gastric cancer cells with peritoneal metastasis, facilitates tumor cell adhesion and invasion within the peritoneal cavity. MMP-9 (41), frequently elevated in gastric cancer patients with peritoneal metastasis, degrades the extracellular matrix, promoting tumor cell migration and metastasis. These biomarkers not only serve as potential tools for early detection of peritoneal metastasis but also offer promising therapeutic targets.

### 3.2 Epidemiological background

Urinary tract obstruction, as an initial manifestation of peritoneal metastasis, is underreported in clinical practice (9, 42). We believe the possible reasons are as follows: 1. Limitations of Imaging: CT is the primary tool for detecting peritoneal metastases (43), but early-stage disease often lacks clear radiological signs, with a reported miss rate of approximately 16% (36, 44, 45). PET/CT or diffusion-weighted MRI have demonstrated superior sensitivity and specificity (46), but our patients refused for personal reasons and economic issues. 2. Low Clinical Suspicion: Typical PM manifestations include ascites, bowel obstruction, and cachexia, which may mask subtle signs of urinary obstruction (22). 3. Epidemiological Data Gaps: Urinary tract obstruction is more common in gynecologic, colorectal, and lymphoid cancers (47). Moreover, most studies on AKI in gastric cancer lack mechanistic stratification (prerenal/renal/postrenal), and hydronephrosis severity is rarely graded or reported, leaving no precise epidemiological data. Using an estimated AKI incidence of 13.9% in gastric cancer and a 5%–10% post-renal cause estimate (48), we calculate post-renal AKI at 0.7%–1.39%. Importantly, the majority of reported gastric cancer-related obstructive uropathy involves distant metastases (e.g., to bladder (8), cervix (9, 10) rather than peritoneal disease causing ureteral compression. Our case likely represents a rare subset within this already small group, consistent with its unreported status.

Analogous cases have been sporadically documented. In a case reported by Elfert et al., a patient was admitted with anuria and bilateral hydronephrosis; CT revealed gastric cancer with PM, and renal function improved after DJ stent insertion (49). Similarly, Boubaker et al. described a patient with PM-induced bilateral ureteral compression and severe hydronephrosis who responded well to DJ stenting (7). We emphasize that although analogous cases of gastric cancer with peritoneal metastasis causing urinary obstruction have been reported, our case is distinct in that only trace hydronephrosis was present initially, leading to clinical underestimation and delayed diagnosis.

### 3.3 Diagnostic pitfalls in minimal hydronephrosis

Hydronephrosis is a sensitive indicator of urinary tract obstruction, reflecting the dilation of the collecting system (2, 50, 51). Ultrasound and CT are key imaging modalities for assessment (52). However, in cases of mild dilation, their sensitivity drops significantly—ultrasound sensitivity can be as low as 6% (7), contributing to diagnostic delays.

Our patient presented with established AKI, but had multiple potential nephrotoxic factors (NSAIDs, cancer-related nephropathy) (2, 5, 16, 53–55), and imaging showed only minimal hydronephrosis. A multidisciplinary team (nephrology, urology, oncology and radiology) reviewed the case and initially ruled out obstruction. After extensive discussion, the patient consented to renal biopsy, which revealed only mild glomerular changes—insufficient to explain the degree of renal dysfunction. This prompted a crucial reassessment: even minimal hydronephrosis can lead to significant AKI.

**Figure 4:**
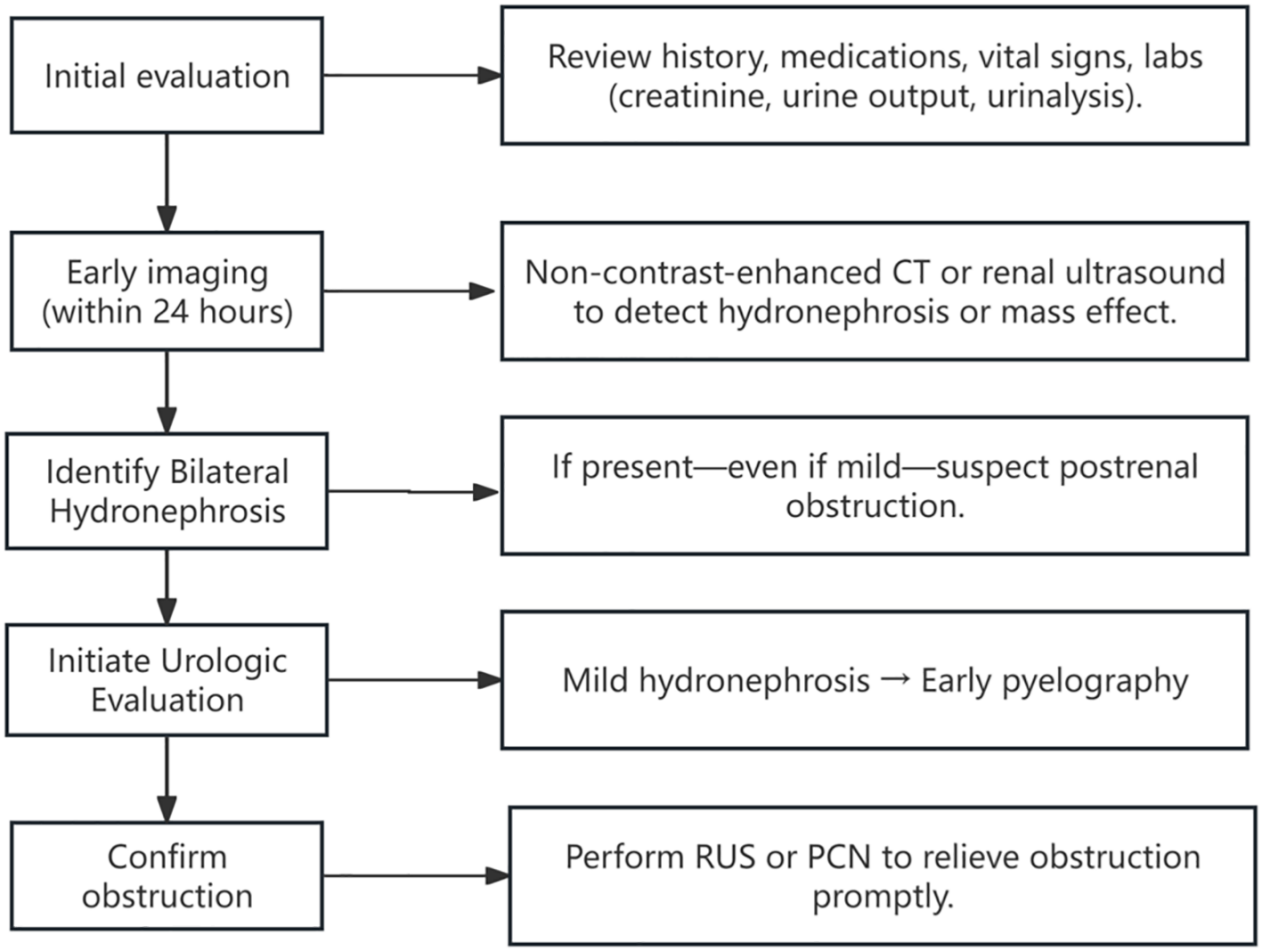
Clinical timeline of the patient’s presentation, diagnosis, and management. Timeline summarizing key clinical events, including serial serum creatinine levels, timing of major interventions (renal biopsy, retrograde ureteral stenting, and percutaneous nephrostomy), dialysis sessions, and clinical milestones such as onset of anuria and hospital discharge. The figure illustrates how initially subtle imaging findings contributed to a delay in diagnosis. Early recognition and prompt relief of obstruction may improve short-term outcomes in patients with malignancy-associated obstructive AKI.

Prior reports have described a rare syndrome—obstructive nephropathy with minimal collecting system dilation (7, 56). These patients exhibit significantly elevated creatinine levels, often accompanied by symptoms such as oliguria/anuria, abdominal pain, and nausea/vomiting, but imaging reveals little to no hydronephrosis. This syndrome comprises 5% of all urinary tract obstruction cases (57). The pathophysiology of this syndrome is likely multifactorial. External compression or fibrosis may impair ureteral peristalsis and disrupt urine flow, leading to renal pelvic pressure and functional decline (58, 59). Yet, in the absence of complete obstruction, the collecting system often lacks significant dilation. Some theories suggest that urine may instead drain via the renal sinus or be rerouted through lymphatic or venous pathways, thereby reducing pelvic pressure and minimizing detectable hydronephrosis on imaging (60, 61). Notably, 60%-66% of these cases result from extrinsic ureteral compression by retroperitoneal fibrosis (RPF) or metastatic malignancy (62, 63). Among them, 86% of AKI cases with negative initial imaging were eventually diagnosed as obstructive through urography (62). Several reported cases well illustrate this phenomenon: Shahzad et al. reported a breast cancer patient with PM presenting with anuric AKI and no visible hydronephrosis (56). AKI was initially thought to be secondary to drug-induced acute tubular necrosis. Due to the application of anticoagulants for deep vein thrombosis, renal biopsy could not be performed. Later, after re-evaluation with pyelography, she was diagnosed with postrenal AKI. Similarly, Onuigbo et al. documented a bladder cancer case where unilateral hydronephrosis was evident, but contralateral obstruction with minimal dilation was only revealed via imaging-guided intervention (64). In other limited reports, obstructive nephropathy with minimal collection system dilation has also been described in colon cancer (65) and uterine cancer (12). All patients had their obstructions relieved through RUS or PCN, resulting in the recovery of renal function. By contrast, our patient had a primary gastric cancer lesion—rarely reported in this context—making this case particularly instructive. These cases remind us that in high-risk populations, early urography should be considered to confirm obstruction, even in the absence of overt imaging findings. Based on these insights, we propose a five-step diagnostic algorithm for postrenal AKI in cancer patients (**Figure 4**).

### 3.4 Therapeutic challenges and multidisciplinary management

The treatment of cancer-related postrenal AKI should emphasize a “rapid assessment–prompt decompression” strategy. In this case, the patient met the KDIGO criteria for stage 3 AKI (66), with acute anuria and hyperkalemia requiring emergent dialysis. After renal biopsy excluded intrinsic renal pathology, follow-up urinary tract ultrasound revealed progressive hydronephrosis, indicating worsening obstruction. Studies have shown that kidney injury due to urinary tract obstruction is partially reversible, and renal prognosis depends primarily on the timely relief of obstruction (5, 67, 68).

After consulting with the urology team, urgent decompression was pursued. Retrograde ureteral stenting (RUS) was selected as the first choice due to its minimally invasive nature, low bleeding risk, minimal impact on daily life, and cost-effectiveness (69–71). In clinical practice, especially in cancer patients, it is crucial to monitor for complications such as obstruction recurrence, stent displacement, or crystallization after double-J stent placement (72–76). A retrospective study reported a 37% failure rate of stent placement in malignant ureteral obstruction, underscoring that RUS alone is often insufficient (77). In our case, immediate improvement in urine output and renal function following stent insertion further confirmed the obstructive etiology. However, the patient developed recurrent anuria four days later. Follow-up abdominal X-ray revealed no twisting or displacement of the stent, we opted for additional percutaneous nephrostomy (PCN). Although ultrasound-guided PCN is considered the gold standard for percutaneous access to the renal urinary system (78), it has limited success in non-dilated systems and carries higher risks of minor vascular injury and bleeding (79). CT, on the other hand, offers more precise visualization of the renal pelvis and calyces, allowing for quicker and safer insertion of the puncture needle (80–82). Thus, CT-guided PCN was performed. The patient, previously anuric, produced 3000 mL of urine within 24 hours post-PCN. Serum creatinine decreased from 632.2 µmol/L to 58.3 µmol/L within 72 hours and remained within the normal range thereafter.

This two-step intervention strategy—RUS followed by PCN—effectively managed the obstruction and highlights the importance of multidisciplinary collaboration and dynamic reassessment in the management of malignant obstructive AKI.

### 3.5 Follow up

Beyond acute management, long-term follow-up is critical to assess prognosis in patients with gastric cancer and peritoneal metastasis. The prognosis of patients with gastric cancer complicated with peritoneal metastasis is generally poor. The median survival period is 4 to 6 months (83), and the 5-year survival rate is usually less than 10% (84). Nevertheless, some patients may benefit from multimodal therapies, including systemic chemotherapy, intraperitoneal treatment, and cytoreductive surgery, along with effective symptom management, leading to extended survival and enhanced quality of life (85–87). Regrettably, two months after discharge, our patient was diagnosed with multiple metastatic lesions. Although an Eastern Cooperative Oncology Group (ECOG) score was not conducted, during this period, she exhibited progressive fatigue and limited activity, and refused further diagnostic assessment or therapeutic interventions, choosing to passively await disease progression.

## 4 Conclusion

In summary, this report describes a rare case of postrenal AKI caused by small dilated obstructive urinary tract lesions caused by gastric cancer involving the peritoneum. In the context of gastric cancer, AKI may act as an early warning signal, prompting a thorough evaluation of the underlying cause and personalized treatment strategies. Initially, the very small amount of hydronephrosis shown on imaging led to an underestimation of obstructive components, and we assumed that the patient’s AKI was attributable to chemotherapy, NSAIDS, and other factors. However, the small glomerular changes found by renal needle biopsy are nonspecific and insufficient to explain severe renal dysfunction. Subsequent recovery of urine volume and renal function after double J tube insertion confirmed obstruction as the main cause. Rapid restenosis (4 days after stent placement) and widespread peritoneal metastasis within 2 months reflect an aggressive clinical course of gastric cancer. Although specific molecular markers, such as HER2 status or MMR deficiency, were not assessed in this case, these features are associated with advanced disease progression in gastric cancer.

This case provides four critical insights for clinical practice and medical education: First, it demonstrates that even mild hydronephrosis may mask life-threatening postrenal AKI in patients with advanced malignancies, necessitating heightened clinical vigilance. Second, it serves as an important teaching example that subtle imaging findings must be carefully evaluated in cases of acute renal deterioration. Third, it highlights how early multidisciplinary collaboration enables timely intervention and may improve short-term renal outcomes. Finally, by challenging the conventional assumption that hydronephrosis severity correlates with degree of obstruction, this rare presentation of peritoneal carcinomatosis contributes valuable new evidence to our understanding of atypical obstructive nephropathy in oncological patients.

## Data Availability

The original contributions presented in the study are included in the article/supplementary material. Further inquiries can be directed to the corresponding author/s.
